# Magnifying Endoscopy with Blue Laser Imaging Improves the Microstructure Visualization in Early Gastric Cancer: Comparison of Magnifying Endoscopy with Narrow-Band Imaging

**DOI:** 10.1155/2017/8303046

**Published:** 2017-08-30

**Authors:** Reiko Kimura-Tsuchiya, Osamu Dohi, Yasuko Fujita, Nobuaki Yagi, Atsushi Majima, Yusuke Horii, Tomoko Kitaichi, Yuriko Onozawa, Kentaro Suzuki, Akira Tomie, Tetsuya Okayama, Naohisa Yoshida, Kazuhiro Kamada, Kazuhiro Katada, Kazuhiko Uchiyama, Takeshi Ishikawa, Tomohisa Takagi, Osamu Handa, Hideyuki Konishi, Mitsuo Kishimoto, Yuji Naito, Akio Yanagisawa, Yoshito Itoh

**Affiliations:** ^1^Department of Molecular Gastroenterology and Hepatology, Graduate School of Medical Science, Kyoto Prefectural University of Medicine, Kyoto, Japan; ^2^Department of Medical Oncology, Fukushima Medical University, Fukushima, Japan; ^3^Department of Pathology and Cell Regulation, Kyoto Prefectural University of Medicine, Kyoto, Japan; ^4^Department of Gastroenterology, Murakami Memorial Hospital, Asahi University, Gifu, Japan; ^5^Department of Surgical Pathology, Kyoto Prefectural University of Medicine, Kyoto, Japan

## Abstract

**Backgrounds:**

Magnifying endoscopy with blue laser imaging (ME-BLI) for diagnosis of early gastric cancer (EGC) is as effective as magnifying endoscopy with narrow-band imaging (ME-NBI). However, there are different EGCs in microstructure visualization between ME-BLI and ME-NBI. This study aimed to clarify the pathological features of the EGCs, in which microstructure visualization was different between ME-NBI and ME-BLI.

**Methods:**

EGCs were classified into groups A (irregular microsurface pattern (MSP) in ME-BLI and absent MSP in ME-NBI), B (irregular MSP in two modalities), or C (absent MSP in two modalities), according to the vessel plus surface classification. We compared the pathological features of EGCs between the three groups.

**Results:**

17, four, and five lesions could be evaluated in detail in groups A, B and C, respectively. Well-differentiated adenocarcinomas with shallow crypts were more frequent in group A than in group B (58.8 and 0%, resp.). The mean crypt depth of group A was significantly shallower than that of group B (56 ± 20, 265 ± 64 *μ*m, resp., *P* = 0.0002).

**Conclusions:**

ME-BLI could better visualize the microstructures of the EGCs with shallow crypts compared with ME-NBI. Therefore, ME-BLI could enable a more accurate diagnosis of EGC with shallow crypts.

## 1. Introduction

Endoscopic submucosal dissection (ESD) for early gastric cancer (EGC) is commonly performed worldwide as a standard therapy [1]. In the Japanese gastric cancer treatment guidelines, pathological diagnosis is essential to decide ESD indication [2, 3].

Image-enhanced endoscopy is performed for the diagnosis of gastric lesions because of its accuracy and applicability. Magnifying endoscopy with narrow-band imaging (ME-NBI) has a high diagnostic accuracy of superficial gastric lesions compared with white-light imaging endoscopy [4–7], because ME-NBI can also visualize the microstructures and microvessels of the EGCs.

Blue laser imaging (BLI) has recently been developed as a combination of two kinds of laser lights for narrow-band light observation. Magnifying endoscopy with BLI (ME-BLI) is useful for evaluating mucosal surfaces, including surface blood vessels and structure patterns [8–12]. We previously reported the usefulness of ME-BLI for the diagnosis of EGC compared with white-light imaging endoscopy [13]. In another study of ours, we reported that there were differences in 15 of 90 EGCs (16.7%) in microstructure visualization between ME-BLI and ME-NBI. In all 15 of those cases, ME-BLI showed irregular microstructures, whereas ME-NBI showed nonvisible EGC microstructures [14]. The discrepancy between ME-BLI and ME-NBI has yet to be investigated. Thus, the aim of this study was to clarify the pathological features of EGCs in which microstructure visualization differed between ME-NBI and ME-BLI.

## 2. Materials and Methods

### 2.1. Materials

We retrospectively analyzed 278 consecutive EGC patients who underwent endoscopic examination with both ME-BLI and ME-NBI prior to ESD at Kyoto Prefectural University of Medicine Hospital between September 2011 and August 2014. All patients provided written informed consent to undergo endoscopic examination by both ME-BLI and ME-NBI.

This study was approved by the Ethical Review Committee of Kyoto Prefectural University of Medicine and was carried out in accordance with the Helsinki Declaration of the World Medical Association. In addition, the current study was registered in the University Hospital Medical Information Network Clinical Trials Registry.

### 2.2. Endoscopic System and Device

All examinations were carried out with ME-BLI and ME-NBI. ME-BLI was performed using an EG-L590ZW endoscope (Fujifilm Co., Tokyo, Japan) with the LASEREO endoscopic system (Fujifilm Co.). ME-NBI was performed using a GIF-H260Z endoscope (Olympus Medical Systems, Tokyo, Japan) with the EVIS LUCERA SPECTRUM endoscopic system (Olympus Medical Systems). A black hood (MB-46, Olympus Medical Systems Co.) was attached to the tip of the endoscope to maintain the focal distance during the procedure. The same endoscopic system settings (BLI: image enhancement mode-A6 and color enhancement mode-C1, NBI: image enhancement mode-B8 and color enhancement mode-1) were used for all examinations. The image enhancement mode and color enhancement mode of the ME-BLI are similar to those of the ME-NBI.

### 2.3. Endoscopic Evaluation

Microstructure visualization of each EGC was evaluated using both ME-BLI and ME-NBI according to the vessel plus surface classification (VSCS). VSCS defined microstructure visualization as a microsurface pattern (MSP), and the findings were defined as regular, irregular, and absent MSP [4]. We classified the lesions into three groups as follows: lesions with irregular MSP on ME-BLI but with an absent MSP on ME-NBI were classified as group A; lesions with an irregular MSP on both ME-BLI and ME-NBI were classified as group B; and lesions with an absent MSP pattern on both ME-BLI and ME-NBI were classified as group C. There were 36 lesions (13%) in group A, 236 lesions (85%) in group B, and six lesions (2%) in group C. There were no lesions with an absent MSP on ME-BLI but with an irregular MSP on ME-NBI.

### 2.4. Pathological Evaluation and Diagnosis

Specimens of all EGCs were resected by ESD. The resected specimens were subsequently fixed with neutral buffered formalin (10% or 20%), sectioned in 2-mm intervals, observed by a stereomicroscope (Nikon SMZ-10, Nikon Corporation, Tokyo, Japan), and photographed by a digital camera system for microscopy (Nikon DS-Fi2-L3, Nikon Corporation, Tokyo, Japan). The sections were embedded in paraffin blocks, sliced into 3-*μ*m-thick sections, stained with hematoxylin and eosin (H&E), and then examined by pathologists. Pathological diagnoses were carried out according to the Japanese Classification of Gastric Carcinoma proposed by the Japanese Gastric Cancer Association [15] by a highly experienced clinical pathologist (A. Y.) who was blinded to the magnified endoscopic findings.

**Figure 1:**
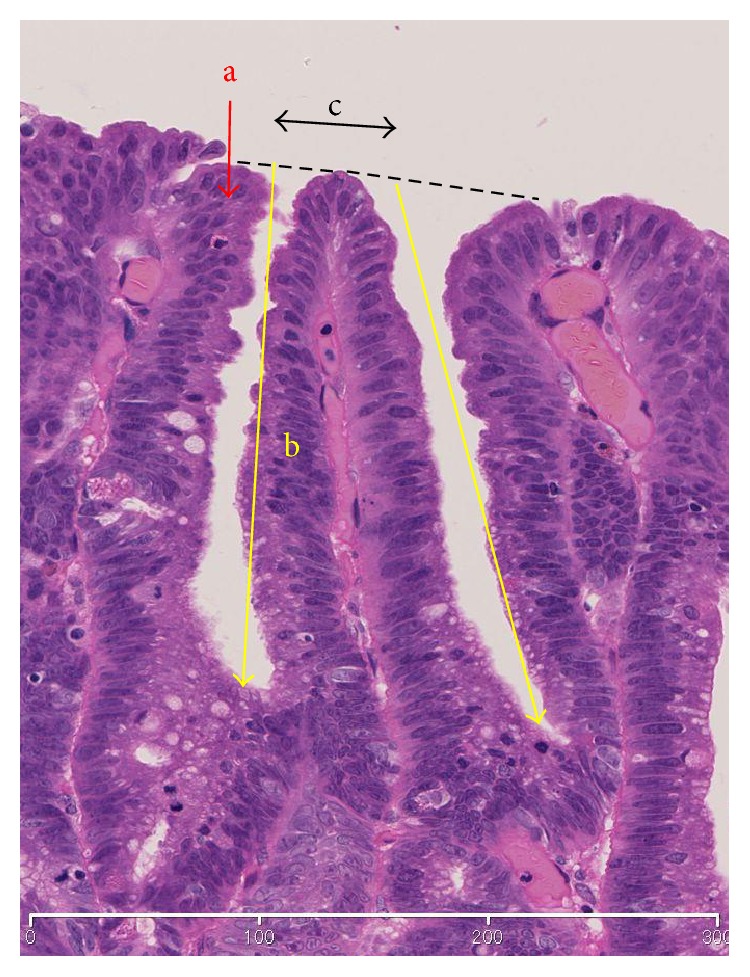
Schema of crypt and epithelium. a, surface epithelium. b, depth of crypt. c, intervening part. Depth of crypt: defined as the distance between the bottom of the crypt and the dotted line that connects the top of the crypt structures.

For all the H&E-stained sections, we confirmed that the histological areas examined one to one corresponded to the parts of images obtained by ME-BLI and ME-NBI. Our experienced clinical pathologist (Y. F.) and endoscopist (R. K.) compared the endoscopic images, stereomicroscopic images, and histological images. The histopathological images that were captured in the virtual slide system (NanoZoomer 2.0-HT, Hamamatsu Photonics, Hamamatsu, Japan) were evaluated using viewer software (NDP.view, Hamamatsu Photonics). We examined the histological type and characteristics of the histopathological findings of each group. Furthermore, in groups A and B, the depths of three crypts in each lesion were measured using the viewer software in accordance with a recent study [5]. Crypt depth was defined as the distance between the bottom of the crypt and the line that connects the top of the crypt structures (Figure 1).

### 2.5. Statistical Analysis

An unpaired *t*-test was used to compare the mean depth of the crypts in groups A and B. The results are presented as mean ± SEM. The Kruskal-Wallis test and *χ*^2^ test were used to investigate the clinicopathological features among the three groups. The criterion for statistical significance was taken as *P* < 0.05. These analyses were performed using the GraphPad Prism 5 program (GraphPad Software Inc., San Diego, CA, USA).

## 3. Results

### 3.1. Clinicopathological Features of Early Gastric Cancers

Seventeen lesions in group A, four lesions in group B, and five lesions in group C could be evaluated in a one-to-one correspondence between the endoscopic image and pathological finding in detail. In groups A and B, all lesions were well- or moderately differentiated EGCs. In group C, there were three poorly differentiated EGCs and two well-differentiated EGCs. The clinicopathological features of three groups are summarized in Table 1.

In group A, ten lesions had shallow crypts, three had a small number of crypt openings, two had curved crypts and long intervening parts, and two had short intervening parts. In group B, all four lesions had deep and straight crypts. In group C, the glandular architecture of three lesions was destroyed due to infiltration of signet-ring cell carcinoma in the entire mucosal layer, one lesion had a very short intervening part, and one lesion had pseudostratified cancer cells (Table 2).

We focused on the ten lesions with shallow crypts in group A and compared their crypt depths with those of four lesions in group B. The mean crypt depth of the group A lesions was significantly shallower than that of the group B lesions (56 ± 20 *μ*m and 265 ± 64 *μ*m, resp., *P* = 0.0002) (Table 3).

### 3.2. Representative Cases

**Figure 2:**
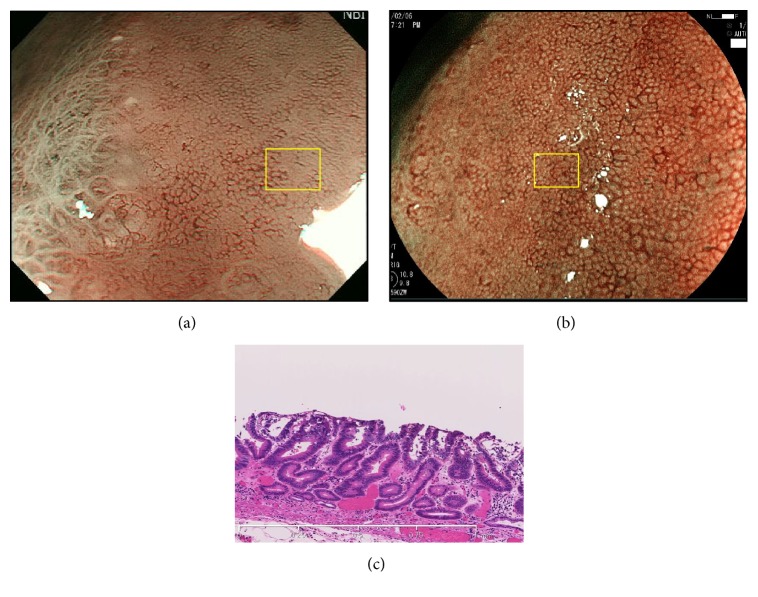
A well-differentiated adenocarcinoma in group A. (a) MSP was absent on ME-NBI (yellow box). (b) ME-BLI showed an irregular MSP (yellow box). (c) Histological view of the yellow box in Figures 2(a) and 2(b). The crypts were very shallow.

**Figure 3:**
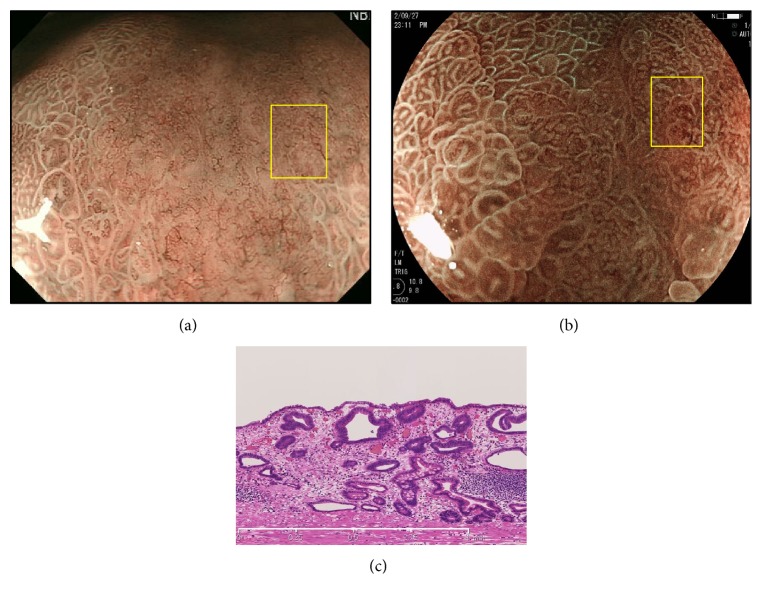
A well-differentiated adenocarcinoma in group A. (a) MSP was absent on ME-NBI (yellow box). (b) ME-BLI showed an irregular MSP (yellow box). (c) Histological view of the yellow box in Figures 3(a) and 3(b). There was a small number of crypt openings in the surface layer.

Representative cases 1 and 2, from group A, are shown in Figures 2 and 3, respectively. Representative case 1 was a depressed well-differentiated adenocarcinoma on the anterior wall of the fornix, and representative case 2 was a depressed well-differentiated adenocarcinoma on the lesser curvature of the upper gastric body. In both cases, MSP was absent on ME-NBI (Figures 2(a) and 3(a)), but an irregular MSP was visible on ME-BLI (Figures 2(b) and 3(b)). Very shallow crypts can be observed in case 1 (Figure 2(c)), and a small number of the crypt openings are visible in case 2 (Figure 3(c)).

**Figure 4:**
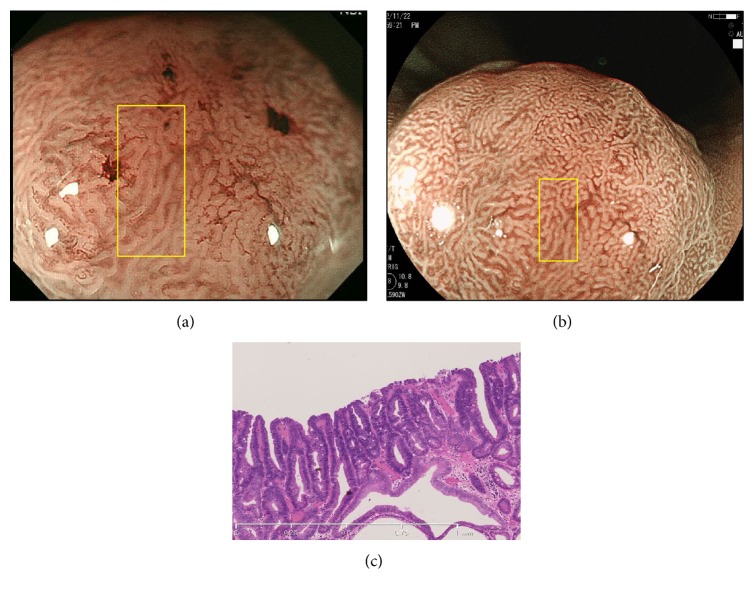
A well-differentiated adenocarcinoma in group B. (a) ME-NBI showed an irregular MSP (yellow box). (b) ME-BLI showed an irregular MS (yellow box). (c) Histological view of the yellow box in Figures 4(a) and 4(b). The crypts were deep and straight.

Representative case 3, from group B, was an elevated well-differentiated adenocarcinoma on the posterior wall of the middle gastric body (Figure 4). The MSP was irregular on both ME-NBI (Figure 4(a)) and ME-BLI (Figure 4(b)). Deep and straight crypts were observed in case 3 (Figure 4(c)).

**Figure 5:**
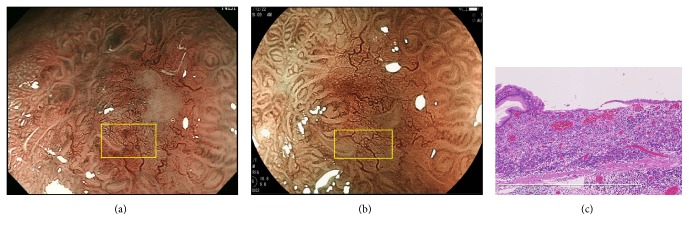
A signet-ring cell carcinoma in group C. (a) MSP was absent on ME-NBI (yellow box). (b) MSP was also absent on ME-BLI (yellow box). (c) Histological view of the yellow box in Figures 5(a) and 5(b). The glandular architecture was destroyed due to infiltration of signet-ring cell carcinoma in the entire mucosal layer.

Representative case 4, from group C, was a depressed signet-ring cell carcinoma on the greater curvature of the lower gastric body (Figure 5). The MSP was absent on both ME-NBI (Figure 5(a)) and ME-BLI (Figure 5(b)). The glandular architecture was destroyed due to infiltration of signet-ring cell carcinoma in the entire mucosal layer. MSP was therefore absent in case 3 (Figure 5(c)).

## 4. Discussion

To our knowledge, this is the first comparative report of pathological features of EGC with different microstructure visualization between ME-NBI and ME-BLI. Yao et al. reported that the feature of MSP was characterized by marginal crypt epithelium (MCE) on ME-NBI. Therefore, MSP findings were useful for diagnosing the histological types of EGC [4]. ME-NBI can typically visualize MSP in differentiated, but not in undifferentiated, EGCs. However, there have been a few cases of differentiated EGCs in which ME-NBI was unable to visualize MSP [16]. Yagi et al. reported that EGCs with a clear white zone, which they advocated as the border of the uniform or heterogeneous MCE structure, tended to have wide intervening parts and deep crypts in the ME-NBI images [5]. The white zone was considered to be synonymous with MSP.

In our study, ME-BLI was able to visualize the MSP of the EGCs with histological features including shallow crypts, a curved crypt with a long intervening part, a short intervening part, and a small number of crypt openings, all of which could not be visualized on ME-NBI. Moreover, the mean crypt depth was 56 ± 20 *μ*m in ten EGCs with an irregular MSP on ME-BLI but an absent MSP on ME-NBI. The mean crypt depth was 81 ± 25 *μ*m in the EGCs where the white zones were nondistinct or invisible on ME-NBI [5]. Therefore, ME-BLI might visualize shallower crypts compared with ME-NBI. The difference in MSP observed by ME-BLI and ME-NBI might have been attributed to the difference in the light used in the two systems. BLI is made by a combination of both 410 and 450 nm lasers [8]. However, NBI is a technique by which spectral features are modified by narrowing the bandwidth of spectral transmittance using filters adjusted to both 415 and 540 nm. Additionally, the bandwidths of BLI are less than 2 nm, which is much narrower than the bandwidth of NBI (30 nm) [17].

It has also been reported that lesions with short intervening parts tended to show a nondistinct or nonvisible white zone [5]. In the current study, there were two lesions with short intervening parts (one in group A and the other in group C). These results show that ME-BLI might visualize shorter intervening parts compared with ME-NBI, but it is difficult to evaluate this accurately due to the small number of such cases in our study.

Currently, ESD is widely performed for EGC, and histological diagnosis prior to ESD is important to decide the adaptation of the ESD criteria. Several studies have reported associations between MS and histological types. Kanesaka et al. reported that MSP was absent in 50% or more of undifferentiated EGCs, and that the endoscopic finding of an absent MSP contributed to a differential diagnosis between undifferentiated EGCs and differentiated EGCs. However, 17% of the cases with absent MSP were well-differentiated adenocarcinomas [16]. It is assumed that these misdiagnostic cases may have included cases with an irregular MSP on ME-BLI, which is similar to those in group A in the present study, since all lesions were differentiated EGCs. It may be possible to reduce the number of false positive cases of undifferentiated EGC by using ME-BLI, due to its excellent capability of visualizing MSP compared with ME-NBI.

Our study had some limitations. First, it was based on a retrospective analysis in a single center. Second, the number of cases that could be precisely evaluated in a one-to-one correspondence between the endoscopic images and the histological images was limited. Third, we evaluated the ME-NBI images obtained from a first-generation NBI system. Currently, a new electronic endoscopy system is available (EVIS LUCERA ELITE CV-290/CLV-290SL; Olympus Medical Systems), and the images obtained using that system may differ from the first-generation NBI images.

## 5. Conclusions

ME-BLI was able to visualize the microstructures of EGC cases with shallow crypts compared with ME-NBI. In addition, ME-BLI could provide a more accurate endoscopic diagnosis of EGC with shallow crypts.

## Figures and Tables

**Table 1:** Clinicopathological features of early gastric cancers.

	Group A	Group B	Group C	*P* value
	*n* = 17	*n* = 4	*n* = 5	
Mean age (range), year	69 (50–81)	75 (64–88)	66 (51–83)	ns
Sex				
Male	15	2	3	ns
Female	2	2	2	
Location				
Upper third	6	0	1	ns
Middle third	6	2	2	
Lower third	5	2	2	
Macroscopic type				
Elevated	5	2	1	ns
Depressed	10	2	4	
Mixed	2	0	0	
Mean tumor size (range), mm	18.4 (6–60)	17.3 (5–36)	17 (8–32)	ns
Pathological diagnosis				
Well differentiated	15	3	2	ns
Moderately differentiated	2	1	0	
Poorly differentiated depth	0	0	3	
m	15	4	3	ns
sm1	2	0	1	
sm2	0	0	1	
UL				
–	15	3	3	ns
+	2	1	2	
Invasion				
–	15	4	3	ns
ly+ or v+	2	0	2	
R0 resection (%)	100	100	80	ns

**Table 2:** Histological features of each group.

	*n*
Group A	
Shallow crypts	10
Small number of crypt openings	3
Curved crypt and long intervening parts	2
Short intervening parts	2
Group B	
Deep and straight crypts	4
Group C	
Signet-ring cell carcinoma in the entire mucosal layers	4
Very short intervening parts	1
Pseudostratified cancer cells	1

**Table 3:** Mean depth of crypts in groups A and B.

	Group A	Group B	*P* value
Crypt depth (*μ*m)	56 ± 20	265 ± 64	0.0002
